# Design of automated training and monitoring system for alcohol-based hand rub surgical hand preparation

**DOI:** 10.1186/1753-6561-5-S6-O32

**Published:** 2011-06-29

**Authors:** S Ameling, GÓ Fearghaíl, S Álvarez, G Lacey

**Affiliations:** 1 Computer Science and Statistics, Trinity College Dublin, Dublin, Ireland; 2 Department of Electronics, University of Alcalá, Madrid, Spain

## Introduction / objectives

Alcohol Based Hand Rub (ABHR) for surgical hand preparation has been proposed and a multi-step procedure was proposed by the World Health Organisation in 2009. This represents a culture change for surgical staff and an automated training and monitoring system supports both the surgical and infection control staff is proposed.

## Methods

The proposed system extends the SureWash automated hand hygiene training system developed in Trinity College Dublin. A larger operating space and a wider range of hand and arm motions are required. Three approaches to system design were taken: conventional single camera (IDS, DE), stereo-camera pair (Point Grey, CA) and a 3D camera (Microsoft, US).

## Results

The cameras were positioned above the users with a clear view of their hands. For the single camera system background modelling and skin detection were combined to find the hands and arms. The solution was effective but was sensitive to the intensity and colour of light in the workspace. The stereo camera system measured the distances to objects in front of the camera and was combined with the skin detection to find the hands and arms. The system performed well but required significant processing power. The 3D camera system could detect the arms well but the camera had to be more than 50cm above the top of the user and multiple systems in a confined space can create interference.

## Conclusion

Three designs were evaluated in the development of an automated training and monitoring system for alcohol based hand rub surgical hand preparation and the next step is to test each in the surgical scrub room and develop an engaging training programme for surgical staff.

## Disclosure of interest

S Ameling: None declared, G. Ó Fearghaíl: None declared, S. Álvarez: None declared, G. Lacey Shareholder of GLANTA Ltd.

